# Angiotensin-converting enzyme inhibitors will not help in improving stroke outcome if given immediately after stroke

**DOI:** 10.4103/0972-2327.70871

**Published:** 2010

**Authors:** Rohit Bhatia

**Affiliations:** Department of Neurology, All India Institute of Medical Sciences, New Delhi-110 029, India

Studies have shown that monotherapy with antiplatelets and angiotensin converting enzyme inhibitors (ACEI) or statins lowers the risk of myocardial infarction or stroke, and there has been interest in combining these therapies for primary and secondary prevention of cardiovascular disease.[1] The renin-angiotensin-aldosterone system (RAAS) has been studied the most over the years and been shown to be involved in blood pressure regulation and other cardiovascular and renal mechanisms.[2] The question of whether the use of ACEI in an acute phase of stroke leads to an improvement in the outcome is challenging. I would look at this concept from two perspectives; one from the concept of lowering blood pressure and the other from a ‘protective’ angle.

Relationship between blood pressure and stroke is well-established from the perspective of causation and recurrence among hypertensive and non-hypertensive subjects, and we all agree to the fact that antihypertensive treatment is an important component in the primary and secondary prevention of stroke.[3,4] However, there seems to be a poor consensus on blood pressure lowering in acute stroke. There is a concern that lowering of BP in the first 24 – 48 hours after acute stroke may reduce perfusion, dampen the collateral circulation, and extend the ischemic region, due to failure of auto-regulation in the injured brain.[5] Lowering of blood pressure in the acute phase has been previously observed to be independently associated with a poor outcome.[6]

Evidence for the benefit of lowering BP in acute phase of stroke is scarce, and death associated with elevated blood pressure, at admission with acute stroke, is not affected by the lowering of BP.[7] Eveson and colleagues[8] examined the safety and efficacy of Lisinopril, starting at 5 mg orally for a week and initiated 20 hours after stroke. Although BP reduction was well tolerated, yet there was no difference in the outcome between the treated and placebo groups. In a larger, randomized double-blind, placebo-controlled CHIPPS trial (*Controlling Hypertension and Hypotension Immediately Post-Stroke*),[9] 179 patients were randomly assigned to receive either labetolol, lisinopril or placebo during the acute phase of stroke. This was followed by chronic treatment with an ACEI with or without a diuretic unless contraindicated. The effect of antihypertensive drugs on early (two-week) death or disability and late (90-day) mortality was assessed. There was no difference in the primary outcome of death or dependency at two weeks between the active versus placebo group. (RR 1.03, 95% CI 0.80 – 1.33; *P* = 0.82), although the three month mortality was reduced (9.7% versus 20.3%). However, the results need cautious interpretation in view of the small number of patients and author’s assumption that ACEI was continued after the acute phase, as details of the prescriptions were not known.[7]

Although a different class of drug was used, the data from the PROFESS[10] (*Prevention Regimen for Effectively Avoiding Second Strokes*) trial is interesting. This trial randomized 20,332 patients to either Telmisartan (angiotensin receptor blocker / ARB) or placebo with a median time from qualifying stroke to randomization of 15 days with nearly 70% of the patients being randomized within 10 days. Overall, there was no significant difference between the risk of stroke recurrence between patients taking Telmisartan and those taking placebo [880 (8.7%) versus 934 (9.2%); difference -54; hazard ratio 0.95 (0.86 – 1.04); 95% CI 0.86, 1.04; *P* = 0.231]. This only changed after about 1.5 years of follow-up, with the risk curves separating in favor of Telmisartan. In fact there was a suggestion that it could be worse in the first six months with a higher number of strokes in the Telmisartan group [347 versus 326; difference + 21; hazard ratio 1.07 (0.92 – 1.25); *P* = 0.042]. In a meta analysis of the PROFESS (PRevention Regimen for Effectively Avoiding Second Strokes) and TRANSCEND[11] studies, Telmisartan had no effect on the composite end point (cardiovascular death, myocardial infarction, and stroke) for the first six months.[3,12] In the often quoted PROGRESS (Perindopril Protection Against Recurrent Stroke Study) trial,[13] 6105 patients with a history of stroke or transient ischemic attack (TIA) within the previous five years were included. The median interval from the qualifying event to randomization was eight months, making this trial ideally unsuitable for this discussion. However, it was interesting to note that the perindopril arm alone had no discernible reduction on the risk of stroke, compared to the combination of perindopril and indapamide. This seemed to be related to a greater BP reduction rather than some other drug effect.

From a protective concept, ACEI have received quite a lot of attention over the last few years. A great deal of enthusiasm was generated based on the results of the HOPE (Heart Outcomes Prevention Evaluation)[14] study, with regard to the possible beneficial properties of these drugs on the endothelium and pathogenesis of atherosclerosis. However, we all know that stroke is an acute event caused by the end result of these factors. Long-term effects of these drugs are still understandable by the analogy of remodeling effects beyond BP control, although, these may not seem reproducible from the perindopril arm alone, as per the PROGRESS study cited earlier. How these drugs would affect the outcome during an acute phase of stroke is not clear, as many important factors contribute, such as, degree, depth, and duration of ischemia; collaterals and recanalization using therapeutic strategies like intravenous rtPA and / or neurointerventional treatment. Studies over the past few years have shown that the cerebrovascular protective effects of antihypertensive treatment may differ according to the characteristics of the drugs used to achieve blood pressure reduction. In many of these studies, however, the magnitude of blood pressure reduction differed in the various treatment groups, making proper interpretation of the data difficult.[15] In the Captopril Prevention Project (CAPPP), for example, the stroke incidence was greater in the group treated with an ACE inhibitor than in the conventionally treated control group, presumably because conventional treatment triggered greater blood pressure reduction.[16] Similar findings were obtained in the Antihypertensive and Lipid-Lowering Treatment to Prevent Heart Attack Trial (ALLHAT).[17] A meta-analysis of ACEI clinical trials suggested that there may not be a blanket ‘class effect’ of these agents and a further comparison of enalapril / lisinopril to perindopril suggested outcomes in favor of perindopril.[18] However, the authors themselves cautioned about the interpretation of these results, as many of the outcome studies used for analysis involved the use of perindopril in varying dosages and in combination with many other drugs, thereby making it impossible to attribute all the benefits achieved, solely to the effects of ACEI. Also, indirect comparisons are prone to bias and the most reliable way to assess whether one ACEI is better than the other is by a direct head-to-head comparison.[19]

There have been studies looking at prestroke use of ACEI and its outcome. Kumar and colleagues[1] showed that a cocktail of three drugs (antiplatelet, statin, and ACEI), if used together, lead to a lower stroke severity at the baseline. However, the NIHSS (National Institutes of Health Stroke Scale) score was significantly lower only in patients who were taking triple therapy with antiplatelet plus statin and an ACEI at stroke onset, in comparison with patients on antiplatelets alone, antiplatelets and either statin or ACEI, or no regimens. However, there was no time vs. group interaction; suggesting that the effect on length of hospital stay and discharge status were related to the differences in initial severity rather than an effect on the recovery process. Chitravas and colleagues[20] also looked at the effect of prestroke use of ACEI on acute stroke severity and death at 28 days. Although the authors showed an independent association of prestroke ACEI use on reducing severe neurological deficits and death; when taking into account the initial NIHSS score, the association between prestroke ACEI use and early death was less impressive. Also, this study was retrospective with small numbers, thereby reducing its strength. In another similar study, among 126 retrospectively analyzed patients, the authors found a reduced baseline mean NIHSS (5.5 vs. 9, *P* = 0.03) among patients taking ACEI prior to there stroke, as compared to the ones who were not. However, no difference in infarct volumes was seen.[21] The study had inherent limitations of being retrospective, non-randomized for treatment allocation, of small sample size, and used an arbitrary cut off of NIHSS scores. Also, no outcome data was presented in this study. Such effects need to be validated in large prospective RCTs (randomized controlled trials), where a direct comparison between ACE and non-ACE regimens is made. Over the years, data has been mainly growing with ARBs (angiotensin receptor blockers) rather than with ACEIs. ONTARGET (The Ongoing Telmisartan Alone and in Combination with Ramipril Global Endpoint Trial)[22] observed the effects of a combination regimen of Telmisartan and Ramipril on patients with vascular disease or high-risk diabetes. Telmisartan was found to be equivalent to Ramipril and a combination of the two drugs was associated with more adverse events, without any increase in benefit.

Thus, literature on ACEIs in the acute phase of stroke is meager and studies looking into long-term outcomes are few and are mainly derived from PROGRESS and HOPE trials.[14] Although these studies suggest benefit in long-term, they do not answer the question of whether early or late treatment with these agents will change the outcome. Although one may assume that they should be started in the acute phase of stroke for the theoretical effects of these drugs on blood pressure, inflammatory cascade, neuroprotection and / or blood pressure reduction, this is yet to be studied systematically and extrapolated from clinical outcomes. Thus at this time, we lack strong evidence about the probable impact of these drugs (especially where there is a doubt about class effect), if started early, on stroke outcomes in the acute or chronic phase.
